# E-Commerce Brand Ranking Algorithm Based on User Evaluation and Sentiment Analysis

**DOI:** 10.3389/fpsyg.2022.907818

**Published:** 2022-06-23

**Authors:** Nie Chen

**Affiliations:** Department of Electronic Commerce, Zhejiang Business Technology Institute, Ningbo, China

**Keywords:** e-commerce, sentiment analysis, brand ranking, consumers, product information

## Abstract

**Objective:**

Consumers often need to compare the same type of products from different merchants to determine their purchasing needs. Fully mining the product information on the website and applying it to e-commerce websites or product introduction websites can not only allow consumers to buy products that are more in line with their wishes, but also help merchants understand user needs and the advantages of each product. How to quantify the emotional tendency of evaluation information and how to recommend satisfactory products to consumers is the research purpose of this paper.

**Method:**

According to the analysis of the research object, this paper uses the Python crawler library to efficiently crawl the data required for research. By writing a custom program for crawling, the resulting data is more in line with the actual situation. This paper uses the BeautifulSoup library in Python web crawler technology for data acquisition. Then, in order to ensure high-quality data sets, the acquired data needs to be cleaned and deduplicated. Finally, preprocessing such as sentence segmentation, word segmentation, and semantic analysis is performed on the cleaned data, and the data format required by the subsequent model is output. For weightless network, the concept of node similarity is proposed, which is used to measure the degree of mutual influence between nodes. Combined with the LeaderRank algorithm, and fully considering the differences between nodes in the interaction, the SRank algorithm is proposed. Different from the classical node importance ranking method, the SRank algorithm fully considers the local and global characteristics of nodes, which is more in line with the actual network.

**Results/Discussion:**

This paper calculates the sentiment polarity of users’ comments, obtains the final user influence ranking, and identifies opinion leaders. The final ranking results were compared and analyzed with the traditional PageRank algorithm and SRank ranking algorithm, and it was found that the opinion leaders identified by the opinion leader identification model integrating user activity and comment sentiment were more reasonable and objective. The algorithm in this paper improves the efficiency of operation to a certain extent, and at the same time solves the problem that sentiment analysis cannot be effectively used in social network analysis, and increases the accuracy of e-commerce brand ranking.

## Introduction

With the rapid development of the Internet, e-commerce sites have become an important channel for users to purchase goods through the Internet, resulting in a large number of online reviews (Ji et al., 2019). As one of the manifestations of online word-of-mouth, online reviews are increasingly important in the field of e-commerce (Scholz et al., 2017). Multiple studies have shown that when consumers are shopping online, the information quality of online reviews is an important factor that affects consumers’ purchasing decisions, as well as the reputation of merchants (Zhang et al., 2018).

In e-commerce websites, more than 60% of the shopping users will write product reviews on e-commerce websites in order to “share shopping experience and help others to buy” (Tang et al., 2018). At the same time, the number of consumers shopping on e-commerce websites has been on the rise. Therefore, the number of user reviews is also increasing rapidly year by year (Fu et al., 2017). For some best-selling products, there are even tens of thousands of reviews. Potential consumers seek user reviews before shopping. When it comes to helping, it may be drowned in the mass of comments and it is difficult to collect really effective information (Shoja and Tabrizi, 2019). In addition, different user reviews focus on different aspects, so the content of the reviews also shows great differences, which also makes it difficult for potential users to obtain comprehensive evaluations of other users on the product when browsing a large number of reviews. Therefore, it is very necessary to automatically process the user comments of e-commerce websites, and to mine the features and viewpoints that users are interested in. In this context, opinion mining technology for user evaluation information emerges as the times require (Dongmin et al., 2018).

Opinion mining is a field of study used to analyze people’s opinions, sentiments, attitudes, and emotions about entities such as products, services, organizations, individuals, etc. Opinion mining technology involves technologies in many fields such as natural language processing, text mining, information retrieval, machine learning, sentiment classification, etc. It can extract entities and opinions from massive Web text information and perform sentiment analysis (Yang et al., 2020).

E-commerce is rapidly evolving to keep pace with the development of the web (Chen et al., 2019a; Han et al., 2021). Its rapid growth has also created a new problem for companies and consumers. This has made it difficult for many companies to survive due to increasing pressure from competition. People face a dizzying array of choices as they search through the millions of items that interest them. The endless flood of content leaves no time to evaluate and compare all the options. Providing more information on the choice of commodities for consumers before they select commodities that meet their requirements increases the burden of information processing (Chen et al., 2018). Therefore, new marketing strategies, such as one-to-one marketing, customer relationship management, etc., have attracted the attention of researchers and practitioners (Hai et al., 2017). The solution to implementing these strategies is to list a series of recommended products for each specific consumer through a recommender system to help them quickly find products that they may be interested in or want to buy.

In general, the ability to generate personalized rankings for users, or guide users to something interesting and useful in a personalized way, is required among the large-scale possible options (Navimipour and Soltani, 2016; Fong et al., 2017; Jiang et al., 2019). The role of ranking systems is evident when the amount of information online grows so rapidly that it is impossible for individuals to sift through it. In many large e-commerce sites, such as Amazon and CDNow the sorting system has become an important part of how it orders a series of products to consumers (Liu et al., 2017b). A consumer’s likely future purchase behavior can be predicted based on the best-selling products on the website, consumer demographic information, or an analysis of the consumer’s past purchase behavior (Zhang et al., 2020).

Applying a high-quality recommendation system to e-commerce, for users, can quickly choose the products they are interested in, making the shopping experience more comfortable; for e-commerce websites, it can improve users’ loyalty to the website and increase the profitability and popularity of the website (Havrlant and Kreinovich, 2017).

In this paper, the SRank unweighted network important node ranking algorithm is applied to the e-commerce ranking system, which not only mines the user’s browsing behavior, but also mines the attributes of the consumers. In addition, in order to further explore the user’s interest, the information sentiment attitudes generated by consumers are further analyzed, and the products with positive emotions of most users are mined. Combined with the browsing behavior of potential users, the e-commerce brand ranking is completed.

## Methods

### Acquisition of Comment Corpus

For the data needed for research, it can be crawled by writing a custom program, so that the data is more realistic and more in line with real life. For different websites, different data types, and different ways of crawling, we should take the best way to get the required data for experimental verification. Now the most used method is the Python crawler library: selenium library, BeautifulSoup library, pyquery library, urllib library, etc. The urllib library is the standard library that comes with Python, and can be used directly without installation. Features include web page requests, response fetching, proxy and cookie settings, exception handling, URL parsing, and more. The functions of other crawler libraries are similar, as long as the appropriate library is selected according to the required data form.

This article uses the fast and effective web crawler technology for data acquisition, and uses the BeautifulSoup library in Python for crawling. By searching for e-commerce products with high sales and comprehensive evaluation, we selected three products: Huawei mate10, Lenovo computer thinkpad, and Sony camera a6300. Then, we use BeautifulSoup in Python to write a program to crawl the rules of Jingdong Mall, filter out pictures, videos and other information, each crawled more than 2,000 pieces of comment text information, and stored the obtained comment text data as a txt file.

### Comment Corpus Preprocessing

The basic work of data mining is the preprocessing of comment corpus. The pre-standardization of comment corpus is the first step in Chinese text mining. Pre-standardization is to delete useless options and meaningless comments in the data set; the second step is to perform word segmentation, part-of-speech tagging, syntax analysis and semantic analysis and other preprocessing, because the data set after preprocessing can better extract the characteristics of text content. When analyzing Chinese characters such as preprocessing, the processing can be transformed into the processing of tagging words or words, and the effect of word segmentation can be achieved according to the results of tagging.

#### Cleaning Comments

Since the data comes from real e-commerce platforms, the data structure is loose and contains a large number of comments with no specific meaning, so comment data cleaning is required. A lot of information in the original review text data is not helpful for the extraction of evaluation objects and evaluation words, and it also seriously affects the results of the following research. Cleaning comment data is to solve data quality problems and make the data more suitable for mining. The result of data cleaning is a series of processing of various dirty data, providing standard, valuable, continuous data, data mining, etc. for data statistics. Its purpose is to delete duplicate information and correct existing errors. There are two main types: one is numeric data, and the other is non-numeric data. The comment text in this article belongs to non-numerical data, and there are a large number of comments with low or even no value in it. If these worthless comment data are also subjected to word segmentation, word frequency statistics and even sentiment analysis, it will cause great harm to subsequent sentiment analysis. If there is a large impact, the analysis results obtained will also have problems, so before using these review text data, it must be cleaned to remove a large number of such valueless review data.

#### Comment Preprocessing

The comment text is divided into words or phrases, and the comment data is uniformly coded and standardized to form a structured data set. There are many different technical software at home and abroad for researchers to use, such as jieba word segmentation, HanLP word segmentation, etc. The Chinese preprocessing tools are shown in Table 1. Among them, Stanford Parser supports syntactic analysis, and NLPIR and Harbin Institute of Technology LTP support semantic analysis.

**TABLE 1:** Chinese preprocessing tools.

Preprocessing tools	Participle	Part-of-speech tagging	Open source/commercial	Named entity recognition	Supported languages
THULAC	Y	Y	Open source	Y	Java, C++
BosonNLP	Y	Y	Commercial	Y	REST
LTP	N	N	Open source	Y	Java
Tencent Wenzhi	Y	Y	Open source	Y	Java, C++
HanLP	Y	Y	Commercial	Y	Java, C++, Python
FudanNLP	Y	Y	Open source	N	REST
Alibaba Cloud NLP	Y	Y	Commercial	Y	Java, C++
Jieba participle	Y	N	Commercial	Y	C++
NLPIR	Y	Y	Commercial	N	Java

This article requires syntactic analysis and semantic analysis, so the LTP language cloud of Harbin Institute of Technology is selected. It has a 98.5% accuracy rate of analysis. It can ensure better follow-up analysis effect, and also facilitate the input of the data format of the model.

This paper uses Harbin Institute of Technology’s Python open source interface pyltp on Python 3.6 to preprocess the cleaned comment data set such as sentence segmentation, word segmentation, part-of-speech tagging, and syntactic analysis. The semantic analysis is implemented by calling the http request method of the Harbin Institute of Technology Language Cloud API. Through a series of preprocessing processes, the user comment data is divided into 18,516 word strings.

### Sentiment Analysis of Short Text Comments

When performing sentiment analysis in this paper, it is no longer necessary to use any sentiment dictionary. Some training data with emotional labels is input into the convolutional neural network model to train the model, and the model can automatically learn emotions.

Second, sentiment analysis is performed using short text reviews, and the sentiment classification labels of short text reviews are assigned to the product features contained in the reviews. This is a sentence-level judgment method that does not cause any loss of original information and fully considers contextual information. It automatically learns the features required for sentiment analysis and no longer requires manual feature extraction.

When performing sentiment analysis on short text reviews, the problem of sentiment analysis is regarded as a binary classification problem, that is, judging whether the short text reviews are positive or negative. Positive means that users are satisfied with specific product features, and negative means users are dissatisfied with specific product features. Some consumers may express their neutral attitude toward product features, such as “the battery is good for use.” We judge it as a negative review during sentiment analysis, and think that if a product feature does not attract customers to make positive reviews, this product feature will be regarded as a negative review.

Before classification, it is necessary to train and test the classifier, and then apply it for short text comment sentiment classification after the classifier achieves a high classification accuracy. The classifier needs to be trained using a supervised training corpus. The supervised training corpus can be obtained either by randomly extracting parts from short text reviews for annotation, or by using existing supervised training corpora. It is best to include domain-related review data in the training data to improve the domain applicability of the trained classifier. The training data is divided into training set and test set according to the ratio of 9:1. After the classification training is completed, the test set is used to evaluate the sentiment classification effect of the classifier.

After obtaining better model test results, the trained classifier can be used to classify the sentiment of short text comments. The short text review data represented by distributed word vectors is input into the classifier, and the corresponding sentiment polarity labels are output. After classification, each short text review is labeled with positive and negative polarity labels.

### SRank Weightless Network Important Node Sorting Algorithm

The LeaderRank algorithm is proposed on the basis of the PageRank algorithm, mainly to solve the problems of the PageRank algorithm’s slow convergence speed and the failure of the algorithm after falling into a hanging node. The LeaderRank algorithm adds a background node through the original network, which is bidirectionally connected to all nodes in the network, thereby improving the connectivity of the network and ensuring the convergence speed and robustness of the algorithm.

The second role of the background node is similar to the random jump probability in the PageRank algorithm. For the PageRank algorithm, the random surfer will visit any other node in the network with a certain probability, while for the LeaderRank algorithm, the node accesses background nodes with a certain probability, and each node has a different probability of accessing background nodes. The higher the degree of nodes, the lower the probability of accessing background nodes, while the probability of background nodes accessing all other nodes in the network is the same, so that different nodes can be distinguished. The core formula of the LeaderRank algorithm consists of two parts:


(1) $$L\,R_{i}\,\left(k\right)=\sum_{j=0}^{n-1}\sum_{k=1}^{k_{c}}{\bar{a}}_{j\,i}\,L\,R_{j}\,\left(k+1\right)$$

(2) $$L\,R_{i}=L\,R_{i-1}\,\left(k_{c}\right)+\frac{L\,R_{g}\,\left(k_{c}+1\right)}{n-1}$$

Among them, *n* is the total number of network nodes (excluding background nodes), and *LR_i_(k)* represents the score of node *v*_*i*_ at time *k*. *k*_*c*_ is the moment when *LR_i_(k)* reaches a steady state, *LR_i_(k_c_)* and *LR_g_(k_c_)* are the scores of the background node *v*_*i*_ and node *v*_*g*_ after the system reaches a steady state, respectively, and *a*_*ji*_ is the element in the basic Google matrix.

#### Node Similarity

Most of the classical node importance ranking methods only consider the global attributes or local attributes of nodes, and do not consider the influence of the interaction between nodes on the importance of nodes. In PageRank and LeaderRank, each node plays an equal role in its followers, but in real life, people are more influenced by people they are more familiar with. In general, the more similar the social circles of two people are, the closer their relationship is.

Based on this, this section proposes the concept of similarity between nodes, which is used to examine the mutual influence relationship between nodes, as a local attribute to examine the importance of nodes. The higher the similarity between nodes, the greater the ability of interaction between nodes.

In a directed social network, the outgoing edge of a node represents the interest direction of the node, and the incoming edge represents a recognition of its interest by other nodes. It can be considered that if the number of nodes pointed to by two nodes is more, their interests are more similar, and the similarity value is higher. Therefore, the similarity of two nodes is not only related to the number of nodes that they point to in common, but also to the number of nodes that point to them in common.

*SIM*_*ij*_ is defined as the similarity between the node *v*_*i*_ and its neighbor node *v*_*j*_ of the network. The higher the similarity between the two nodes, the higher the degree of mutual influence between the nodes.


(3) $$S\,I\,M_{i,j}=\left(1-\alpha_{i}\,\gamma \right)\,k_{i,j,i\,n}+\gamma \,k_{i,j,o\,u\,t}$$

Among them, α*_*i*_* is the neighbor node of node *v*_*i*_; 0 < γ < 1, when γ = 1/2 can be used to calculate the similarity between undirected network nodes.

#### SRank Algorithm Model

The node similarity considers the local attributes of the nodes, and the global attributes of the nodes are calculated by the LeaderRank algorithm. The SRank (Similarity Rank) algorithm is proposed by combining the local attributes and global attributes of the nodes. The importance value of node *v*_*i*_ is as follows:


(4) $$S\,R_{i}=S\,I\,M_{i\,j}\,L\,R_{i}\,\prod_{j\to \alpha_{i}}\left(1-\gamma \right)\,L\,R_{j}$$

In the node pair, the node with a large degree has a small contribution to the other party. Therefore, adding the degree value of the node, the importance value of the node *v*_*i*_ is obtained as follows:


(5) $$S\,R_{i}=\gamma \,L\,R_{i}\,\prod_{j\to \alpha_{i}}\left(S\,I\,M_{i\,j}\,L\,R_{j}/k_{i,o\,u\,t}\right)$$

The node importance value obtained by the above formula takes into account the different interactions between nodes when the similarity is the same. However, when the similarity between nodes is 0, the SR value of the node is also zero, which is obviously unreasonable. As long as the node pair has connected edges, the node pair will have mutual influence. Based on this, the above formula is modified. *SR*_*i*_ is defined as the final importance of node *v*_*i*_. The final importance value *SR*_*i*_ of node *v*_*i*_ is:


(6) $$S\,R_{i}=L\,R_{i}\,\prod_{j\to \alpha_{i}}\left[S\,I\,M_{i\,j}\,L\,R_{j}\,\left(1-\gamma \right)/\left(k_{i,o\,u\,t}+1\right)\right]$$

## Results

### Pre-ranking of User Activity of E-Commerce Brands

**FIGURE 1:**
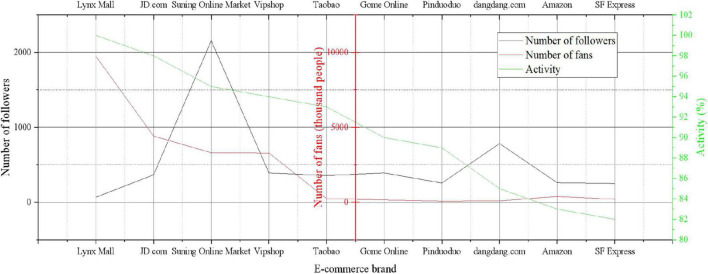
Ranking of e-commerce brand user activity.

According to the attention relationship of e-commerce brand users, the attention matrix of e-commerce brand users is obtained. If e-commerce brand user A follows B, the value in the matrix is 1; if e-commerce brand user A does not follow B, the value in the matrix is 0. This paper uses the MATLAB tool to implement the calculation of the SRank algorithm, assigns the AR value according to the attention matrix, and obtains a stable AR value after 60 iterations. According to the SRank algorithm, the activity ranking of e-commerce brand users can be obtained. After the ranking of SRank values, the evaluation of the influence of e-commerce brand users is no longer limited to the link-in and link-out relationship between e-commerce brand users. The pre-ranked top 10 e-commerce brand users are shown in Figure 1.

In the list of the top 10 e-commerce brand users by e-commerce brand user activity, the first e-commerce brand is “Tmall Mall,” which has the highest number of fans. Its SRank value is much higher than other e-commerce brand users, and its activity value is the highest. The e-commerce brand user “Pinduoduo” has fewer followers than other e-commerce brand users. But it is more active, so it ranks higher than the following e-commerce brand users with more followers.

### Comment Sentiment Polarity Value Calculation Results

This paper uses the Chinese lexical analysis system ICTCLAS of the Chinese Academy of Sciences to segment comments and mark parts of speech, mark the positions of sentiment words, degree adverbs and negative words, and calculate the sentiment polarity value of each comment according to the sentiment polarity calculation rules of polar phrases. The calculation is carried out, and the specific emotion polarity rules are shown in Table 2.

**TABLE 2:** Sentiment calculation rules for polar phrases.

Polar phrase	Calculation formula	Polarity value	Example sentence
Phrase = deg + neg − emo	E(emo) × E(neg) − (1 − E(emo)) × D(deg)	–0.8911	This mask is very hydrating
Phrase = deg + 2emo	E(emo) − (1 − E(emo)) × D(deg)	0.8404	This mask works great
Phrase = deg − neg + emo	E(emo) × E(neg) + (1 − E(emo)) × (D(deg) − 0.1)	–0.6503	This mask is not very hydrating
Phrase = neg + emo n(neg)	E(emo) × E(neg)	–0.7502	This mask doesn’t work
Phrase = emo	E (emo)	0.6909	This mask works well
Phrase = neg − emo n(neg)	E(emo) × E(neg)/E(neg)	0.4302	This mask is not bad

In the process of calculating the sentiment polarity value of comments, first we calculate the sentiment polarity value of each polarity phrase according to the calculation rules shown in Table 2; then we use the sentiment polarity phrase as the basic unit to calculate the sentiment polarity of each comment. We judge whether the sentiment tendency of each comment is positive or negative; then take the comment as the basic unit, and calculate the overall comment sentiment polarity value of each note; finally, take the notes published by the user as the unit, and get the overall comment sentiment polarity value of the user.

Taking the user “JD Jingdong” as an example, after deleting meaningless comments, there are a total of 560 comments in 10 notes. After the comment segmentation and part-of-speech tagging, the sentiment polarity values of sentiment polarity phrases in each comment are calculated according to the rules in Table 2. The comments contain some unsentimental neutral comments, such as “where can I buy it?,” “seeking a link” and other comments expressing interest in the product and willingness to buy, although these comments are not directly expressed in the text. However, it expresses interest in the products recommended by users, and reflects the support and recognition of users on the other hand, so these comments are manually classified as positive emotional comments. The specific sentiment polarity of comments for each note is shown in Table 3.

**TABLE 3:** Sentiment polarity in the user’s “Jingdong” notes.

Comments	Comment sentiment polarity value	Negative comments	Positive comments	Number of comments
Recently, Beauty Potato has encountered a problem: although lipsticks are planted every day, they are all big names, and the wallet can’t afford it!	0.8807	4	117	121
Beauty Potato has always had an immature wish: “I hope all the little sweet potatoes will become makeup bloggers!”	0.9603	2	18	20
This color test video is really invincible!	0.9521	7	79	86
There are constantly babies beating in the background “Why haven’t the Japanese and Korean chapters been published yet!”	0.9713	1	39	40
Beauty Potato, who is usually in charge of helping you to become beautiful, has recently received a new beauty task.	0.9804	0	31	31

**FIGURE 2:**
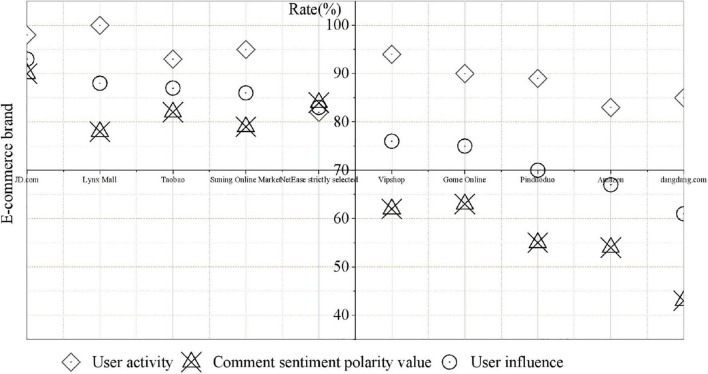
User influence ranking.

The sentiment polarity value of comments is calculated for each user in the top 10 pre-sorts, and the final user influence is calculated after the sentiment polarity value of user comments is obtained. The final ranking of user influence is obtained, and the opinion leaders of the e-commerce brand section are identified, as shown in Figure 2.

According to the ranking of user influence in Figure 2, it can be found that there is a large difference in the sentiment polarity values of different users’ comments. Among them, the user with the highest comment sentiment polarity value is “JD Jingdong” with a value of 0.90, and the user with the lowest comment sentiment polarity value is “Dangdang” with a value of 0.43, a difference of 0.47. Most of the comments published on the “JD Jingdong” platform are dry goods, the content is of high quality and has been well received by everyone. Although “Dangdang” is also very active and has published a lot of comments, most of them are short in length, the quality is not very high, and there are few positive comments, so the comment sentiment value of this platform is relatively low. Therefore, through the sentiment analysis of comments, it is possible to judge the support and approval of other users for the user, so sentiment analysis can further improve the accuracy of opinion leader identification. The comment sentiment polarity value does not appear negative because the opinion leaders are pre-screened in the activity ranking, excluding low-quality users.

### Comparison of Sorting Algorithms

**FIGURE 3:**
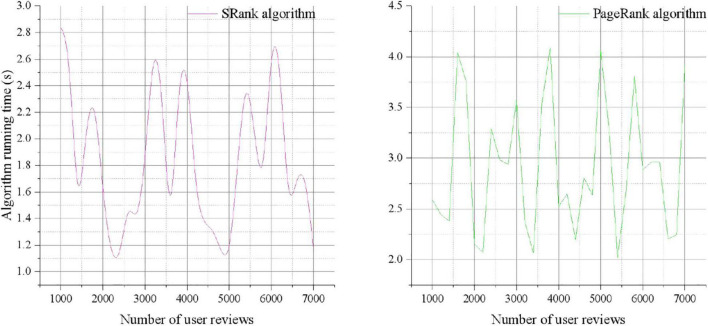
Comparison of real-time ranking between AR algorithm and traditional PR algorithm.

In order to verify the effectiveness of the social e-commerce user activity algorithm, the results of the SRank algorithm are compared with the time consumption of the traditional PageRank algorithm, as shown in Figure 3. This comparison method is chosen because the PageRank algorithm only measures the influence of users based on the attention relationship between users, and the energy consumption of comparing the two rankings can intuitively see the impact of users’ comments on ranking.

From the above experimental results, it can be found that the SRank ranking algorithm in the pre-ranking comprehensively considers the user’s behavior on the social network, so that the identification of opinion leaders in the subsequent stage is more objective and reasonable. It is worth noting that among the calculated top 10 users, the number of fans is not the main factor for user activity. For example, the number of fans of “Pinduoduo” is relatively small, but the final ranking is higher, which shows that the number of fans is related to the user’s performance. On the one hand, there may be some zombie fans among fans, and these users themselves are not active; on the other hand, some fans may have little interaction with him, and did not pay attention to his published content and interact with him in time.

Users with a high number of fans may not be highly recognized and forwarded by other users. The number of fans has nothing to do with the user’s influence, but more of the user’s popularity. Therefore, there is a limitation in measuring a user’s influence solely from the number of fans, and the calculation of other behavioral characteristics of the user can make up for this limitation to a certain extent.

### Comparison of Pre-sorting and Final Sorting

**FIGURE 4:**
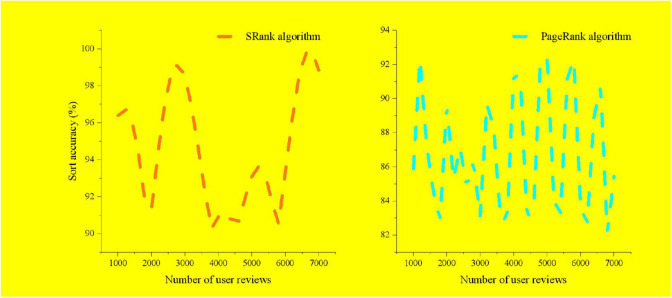
Comparison of the accuracy of pre-sorting and final sorting.

After the calculation of user activity and comment sentiment polarity, the final ranking of user influence is obtained. This paper compares the accuracy of the final results with the pre-sort results, as shown in Figure 4.

In the final ranking of opinion leaders identification, both the user’s activity and the comment sentiment polarity are indispensable. When user activity and comment sentiment are comprehensively considered, the final ranking will be affected. If the identification of opinion leaders is only measured from one aspect, the results are easily affected by unilateral factors, which makes the results biased.

From the perspective of social network structure and comment sentiment, the SRank ranking algorithm builds an opinion leader identification model by integrating user activity and user comment sentiment polarity, so as to identify opinion leaders in social e-commerce, measure the user’s behavioral characteristics from multiple perspectives in the user activity, and more comprehensively express the content of the user’s activity.

User comment sentiment expresses the emotional attitude of other users to the user. For opinion leaders in the community, getting positive support from other users in the community is a manifestation of its importance. Therefore, this paper comprehensively compares the results between the traditional sorting algorithm and the SRank sorting algorithm. The SRank sorting algorithm evaluates user influence from multiple dimensions, avoids the singularity of the identification method of opinion leaders, and makes the model more suitable for the use scenario of social e-commerce.

## Discussion

### Trace the Real Purchases and Benefits Generated by User Reviews

A phenomenon that often occurs in real online shopping is that consumers who have purchase intentions decide to purchase the product after browsing and reading multiple reviews (Andrienko et al., 2021). In this process, the user’s intention is likely to gradually turn into actual purchase behavior through online reviews. One or several online reviews have played a very important role in promoting, but the users who wrote these reviews cannot know that they have great follow-up to written reviews (Guo et al., 2017; Liang and Wang, 2019). In fact, the platform can consider developing a system to realize the retrospective function of online review users and directly connect online reviews and purchase behaviors. Specifically, after a potential user sees the third review of a product, he/she decides to buy the product. At this point, instead of returning to the purchase page, a button can be provided to jump directly to the purchase page (Luan et al., 2017). At the same time, the system could keep a record that the purchase came from the third review, not the fifth or sixth. The system can choose to push this information to the user who wrote the third review, and provide that user with a certain reward. Compared with the simple direct reward incentive method, this can also be an effective method to motivate users to write high-quality reviews, because the effect of online reviews is known at this time, and users who write reviews can clearly know that they’s reviews generate real revenue for the business (Majumder et al., 2017; Pal and Pal, 2018; Fu et al., 2019).

### Establish a Review Classification and Sorting Method Oriented to User Needs

At present, the comments of most e-commerce platforms will be classified by labels, such as positive, moderate, and negative comments, or sorted in chronological order. This classification and sorting method is still limited and cannot comprehensively cover users (Bandhakavi et al., 2017; Chen et al., 2019b). On the one hand, it is difficult for users to effectively evaluate the quality of online reviews they read. On the other hand, users have different needs for review information. Online reviews should be classified according to the content of the reviews and the different perceptions of users, rather than solely according to the sentiment polarity and time of the reviews. Therefore, a more complete review classification and sorting method should be established for user needs (Manek et al., 2017). E-commerce platforms can develop an online review system, which can automatically filter and screen poor quality reviews according to the different characteristics of online reviews to ensure users have a comprehensive understanding of the product. At the same time, more classification criteria are added to distinguish the comment information suitable for different user groups through the system. For example, if a user has a high degree of involvement in the product, he should select high-quality reviews that meet his needs according to its characteristics (Chen et al., 2017). There is no completely uniform standard for evaluating the quality of online reviews. According to the different emphasis in different situations, e-commerce platforms should fully understand the psychological characteristics of consumers and design a better way of presenting information, so that consumers can more accurately and conveniently understand the attributes of products, and display high-quality online reviews for users in a personalized and comprehensive manner (Arulraj et al., 2018; Bouazizi and Ohtsuki, 2019).

### Utilize Seller Responses for Proper Review Management

Many platforms have the function of seller reply, but the use of this function is not very comprehensive (Fong et al., 2017). Some sellers respond very quickly to reviews. But in most cases, they won’t respond.

For some negative reviews, the moderate response from sellers plays a very important role (Chen et al., 2020). When online shoppers saw online sellers’ explanations for negative reviews, their purchase intentions increased significantly.

Therefore, in this case, merchants should have the courage to face negative reviews and respond appropriately (Ji et al., 2019). In addition, sellers should pay more attention to high-impact negative reviews, and managers should do their best to detect and correct negative issues mentioned in negative reviews.

Users who are highly involved in the product also value the seller’s reply. Compared with search-based products, due to the uncertainty of users about experience-based products, experience-based products are more susceptible to negative reviews before experiencing the real product, and platforms should pay more attention to negative reviews of such products (Mittal and Vetter, 2016). Some businesses are nervous about negative reviews, but in fact, negative reviews also need to provide more evidence and clues to prove. With the increase of malicious and false comments on the Internet, users are wise and vigilant in their judgment of online information. Only high-quality negative information that has been demonstrated and confirmed in many ways will be authenticated and affirmed by users. Low-quality negative reviews do not require too much interference for some “unreasonable negative reviews” (Liang and Wang, 2019). Therefore, some reviews require merchants to reply, while others do not require too much intervention. Merchants can make full use of the seller’s reply function for proper review management.

### Promote the Conversion From Online Review Information to Purchase Behavior

Whether it is to motivate the platform to generate high-quality reviews or manage reviews, e-commerce platform managers hope that reviews can provide users with information services and become another channel to promote product sales (Shi et al., 2017; Peng et al., 2018; Xu et al., 2020).

#### Meet the Information Requirements of Users With Different Degrees of Involvement

Users aren’t just looking for a review, they’re looking for the reason behind the review to support their purchase. Low-involvement users are general potential users, high-involvement users are potential users with a high purchase probability, and high- and low-involvement user groups have different emphasis in different situations of involvement (Tang et al., 2018; Hameed and Garcia-Zapirain, 2020). On the one hand, we try our best to convert low-involvement users into high-involvement users; on the other hand, we try to convert high-involvement users into actual consumer users, that is, to maintain potential user groups (Ji et al., 2017). The research results of this paper show that, relatively speaking, the online comment information of the platform is more suitable for low-involvement user groups to read, and it is not enough for user groups with high-involvement requirements (Zhang and Zhong, 2019; Yin et al., 2020). Therefore, it is particularly important for the platform to generate more online reviews that high-involvement users consider to be of high quality. The platform should pay attention to user groups with different degrees of involvement, meet the information needs of users through the quality of comment information, and try to promote users to convert into real buyers (Chen et al., 2019b).

#### Improve User Information Quality Perception and Satisfaction

Users prefer to use appropriate diagnostic product information to reduce uncertainty before making a final purchase decision (Liu et al., 2017a). Merchants should show users reviews that they think are of high quality based on some of the user’s preference characteristics, and improve users’ purchase intentions and final purchase behavior through the first few reviews that are decisive for purchase (Zhang et al., 2018, 2020). Therefore, users’ perception of information quality should be improved. On the other hand, merchants should also strive to improve consumers’ satisfaction with products and business information in online reviews that can stimulate purchase intentions. For example, this study shows that improving social presence can be an effective way to improve satisfaction (Wang et al., 2018).

## Conclusion

From the perspective of comment sentiment, e-commerce brand opinion leaders usually get more positive comment information, which indicates the approval and support of other users. For the brand side, such opinion leaders can more influence other users’ views on the product. Therefore, brands can cooperate with opinion leaders, for example, they can give opinion leaders some products to try out, and after the opinion leaders use it, they can give feedback to other users about their usage experience, so as to transmit positive information about the product to other users. E-commerce companies that cooperate with opinion leaders can reduce advertising expenditure to a certain extent, and choosing the right opinion leaders can help precision marketing, increase the purchase probability of potential consumers, and improve the income of e-commerce companies. At the same time, for social e-commerce platforms, the influence of community opinion leaders on other users’ consumption decisions can be used to divert traffic to the e-commerce platform, which better integrates the community and e-commerce, thus providing a better solution for e-commerce.

## Data Availability Statement

The original contributions presented in the study are included in the article/supplementary material, further inquiries can be directed to the corresponding author.

## Author Contributions

NC researched e-commerce theory and presented e-commerce brand ranking algorithm. The author confirms being the sole contributor of this work and has approved it for publication.

## Conflict of Interest

The author declares that the research was conducted in the absence of any commercial or financial relationships that could be construed as a potential conflict of interest.

## Publisher’s Note

All claims expressed in this article are solely those of the authors and do not necessarily represent those of their affiliated organizations, or those of the publisher, the editors and the reviewers. Any product that may be evaluated in this article, or claim that may be made by its manufacturer, is not guaranteed or endorsed by the publisher.
